# Automatic Segmentation and Recognition of the Microstructure of High-Strength Low-Alloy Steel

**DOI:** 10.3390/ma19122554

**Published:** 2026-06-12

**Authors:** Lu Wang, Ziying Ren, Baoyu Song, Bing Wang, Qiaochuan Chen, Jingjing Wang, Tianpeng Zhou, Yuexing Han

**Affiliations:** 1 School of Computer Engineering and Science, Shanghai University, 99 Shangda Road, Shanghai 200444, China; wanglu@shu.edu.cn (L.W.); bingbignwang@shu.edu.cn (B.W.); qcchen@shu.edu.cn (Q.C.); 2 Ansteel Beijing Research Institute Co., Ltd., Future Science City, Changping District, Beijing 102209, China; mir2014@foxmail.com (Z.R.); sbyllb@sina.com (B.S.); wangj_0388_jwang@163.com (J.W.); ztp1229@163.com (T.Z.)

**Keywords:** superpixel segmentation, grain segmentation, grain extraction, grain identification

## Abstract

Metallographic microstructure analysis is essential for understanding the evolution of steel microstructures during heat treatment and mechanical processing. However, accurate analysis of optical micrographs remains difficult because of blurred grain boundaries, grayscale inhomogeneity within grains, and irregular grain morphologies. To address these issues, this work proposes an automated metallographic image-processing method based on superpixels, DPSS (dual-phase steel segmentation), with the main contribution focused on microstructure segmentation. First, image contrast and boundary visibility are enhanced by edge detection and sharpening. Then, superpixel segmentation is combined with extracted edge information to improve boundary localization and preserve irregular grain morphology, enabling more complete extraction of grain or particle regions from optical images. The proposed method is validated on optical micrographs of Mn-Si low-alloy steel, and the results show that it provides more accurate and complete segmentation than conventional ImageJ (Version: 1.54f)-based processing. Based on the segmented regions, a lightweight neural network is further used for phase identification. The final classification recognition accuracy can reach 99.91%. This classification result serves to demonstrate that the improved segmentation results can provide more reliable inputs for subsequent microstructure recognition. Overall, the proposed method offers an effective and automated solution for metallographic image segmentation and supports more accurate downstream phase analysis.

## 1. Introduction

High-strength low-alloy steel has excellent mechanical properties such as ultra-high strength and plasticity, excellent energy absorption, and good deformability. In practical applications, major advanced high-strength steels are used in body manufacturing to achieve both lightweight and stability of the vehicle and passenger safety. In addition to strength and ductility, modern industrial applications also require a deeper understanding of the wear mechanisms of low-alloy steels. The tribological characteristics and wear resistance of these steels are closely related to their microstructural state. Therefore, microstructural analysis is directly linked to the performance of components exposed to tribological loads, and precise phase identification is important for optimizing wear resistance in service [1]. The microstructure of metals and their content can reveal important information about mechanical properties such as strength, flexibility, yield stress, tensile strength, hardness, surface roughness, and so on [2,3]. Variations in grain size, grain morphology, and phase fraction may lead to significant changes in the final performance of the material. For example, Liu et al. [4,5,6] conducted a more systematic study on the tissue phase transformation law and mechanical properties of 300 M steel by forming 300 M steel block specimens with laser coaxial powder feeding. The tempering temperature affects the orientation of the crystal lattice in 300 M steel, as well as the martensite and bainite, but it is the phase transformation of the martensite that has the most significant effect, precipitation hardening following quenching and tempering can reach 1966 MPa. Calcagnotto et al. [7] investigated the effect of grain refinement on the mechanical properties of dual-phase steels by preparing fine-grained (2.4 μm) and ultrafine-grained (1.2 μm) dual-phase steels by thermal deformation with large strain and annealing in the critical zone, and testing their tensile properties and impact toughness in comparison with coarse-grained (12.4 μm) dual-phase steels by thermal deformation, respectively. Grain refinement increased the initial strain hardening rate and impact toughness of the duplex steel, and the impact fracture of the duplex steel gradually showed a plastic fracture mode with the gradual grain refinement.

Grain refinement is one of the most important strengthening paths of the steel heat treatment. Therefore, grain size estimation is essential in metal property analysis. Furthermore, automatic segmentation of the metallographic grain image is an important step for grain classification [8]. The segmentation method directly affects the final results of the subsequent qualitative metallographic analysis. However, metallographic image segmentation is influenced not only by image noise and grayscale variation, but also by metallurgical factors such as grain-boundary inclination, intragranular grayscale inhomogeneity, and boundary precipitates, which increase the difficulty of accurate grain-boundary extraction. Digital image-processing methods are commonly used in metallographic grain segmentation. For metallographic image segmentation, existing methods can generally be divided into rule-based methods and learning-based methods [9]. Rule-based methods usually determine thresholds according to the grayscale difference between grain interiors and grain boundaries in metallographic images, or directly use edge information to extract grain-boundary contours. Common methods include adaptive thresholding [10] and maximum interclass variance (Otsu) [11]. These methods often achieve good segmentation results for metallographic images with clear grain boundaries and relatively uniform intragranular grayscale distribution. For example, Sun et al. [12] combined dual-thresholding and morphological operations for optical metallographic image segmentation, and obtained good results when the grain boundaries in the experimental images were relatively clear. However, for metallographic images with blurred grain boundaries, non-uniform grayscale distribution within grains, or weak contrast between grain interiors and grain boundaries, it is often difficult to determine an appropriate threshold, which affects the stability and accuracy of segmentation results.

In addition to threshold-based segmentation, edge-based segmentation methods are also commonly used in metallographic image processing. These methods usually first convert the original metallographic image into an edge image, and then reconstruct grain-boundary contours based on the extracted edge information. Campbell et al. [13] proposed a cascade method including filtering, watershed transformation, and region merging for segmenting microstructures in scanning electron microscopy (SEM) and optical microscopy images. This method can generate relatively closed contours, but it is prone to over-segmentation when grain boundaries are complex or local contrast is insufficient. Han et al. [14] proposed a region-clustering segmentation method combining mean shift and flow-based difference-of-Gaussians (FDOG) under conditions of limited material image samples, and achieved a segmentation accuracy of about 87%. Overall, rule-based methods are highly sensitive to image quality and grain-boundary clarity. When metallographic images contain weak grain boundaries, precipitate interference, or irregular grain morphology, their segmentation performance is often limited.

Learning-based methods include machine learning methods and deep learning methods. Compared with rule-based methods, these approaches can automatically learn texture, grayscale, spatial, and morphological features from metallographic images and use classifiers or deep networks to achieve grain or phase recognition and segmentation. Common classifiers include support vector machines (SVM) [15], multilayer perceptrons [16], neural networks [17], optimum-path forests [18], and random forests [19]. For example, Bulgarevich et al. used a fast random forest to automatically segment ferrite, pearlite, bainite, and martensite microstructures [19]. Han et al. [20] proposed a machine learning-based “center-environment segmentation” (CES) feature-learning model, which introduced domain knowledge as pixel features and adopted an iterative machine learning strategy to train and calibrate the segmentation model, achieving good results on various material images, with a segmentation accuracy of about 83%. In deep learning, Choudhury et al. combined watershed segmentation with convolutional neural networks (CNNs) for phase identification in steels [21]. Li et al. [9] proposed a richer convolutional feature (RCF) architecture based on multi-task learning for grain-boundary detection and segmentation in metallurgical images, with a segmentation accuracy of about 91%. Although learning-based methods generally achieve better segmentation accuracy than rule-based methods, their application to metallographic images is still limited by the scarcity of labeled data and the difficulty of pixel-level annotation. This is especially true for optical metallographic images with blurred grain boundaries, uneven intragranular grayscale, and irregular grain morphology. Therefore, a segmentation method that can preserve weak grain-boundary information while reducing over-segmentation is still needed for reliable microstructural characterization and phase identification.

In this study, we propose DPSS (dual-phase-steel-segmentation), a segmentation framework for optical micrographs of high-strength low-alloy steel. The framework is developed to improve the extraction of reliable grain-boundary and phase information from images affected by blurred boundaries, uneven intragranular grayscale, and irregular grain morphology. Unlike conventional ImageJ method, morphology method, and edge-based method, the proposed approach introduces linear spectral clustering into metallographic image segmentation and further combines it with a conditional region-merging strategy to suppress over-segmentation and recover more complete grain regions. In addition, rather than considering segmentation as an isolated task, this study further evaluates the usefulness of the segmented regions for subsequent microstructure recognition. In this way, the proposed method provides a practical basis for quantitative metallographic characterization and subsequent investigations of material performance.

## 2. Dataset

### 2.1. Experimental Materials

In the work, the microstructure of dual-phase (DP) steel consists of austenite and martensite, which is produced by temperature-induced martensite transformation in austenite stainless steel. Such a dual-phase microstructure is more complex than usual DP steel (ferrite and martensite [22]), representing a bigger challenge for accurate microstructural analysis. As an advanced high-strength steel, dual-phase steel has a two-way structure of ferrite and martensite, which provides good strength plasticity, low yield ratio, and high work hardening rate. It is extensively employed in the production of high-strength steel.

The annealing process of cold-rolled dual-phase steel in the two-phase zone will have a significant impact on its microstructure and mechanical properties. The dual-phase steels in the sample were subjected to heat treatment at temperatures of 740 °C, 760 °C, 780 °C, 800 °C, 820 °C, 840 °C, 860 °C, and 900 °C in the two-phase zone. After annealing in the two-phase zone, the cold-rolled dual-phase steel forms ferrite and martensite structures.

The microstructure of dual-phase steel exhibits significant differences at different annealing temperatures. With the increase of continuous annealing temperature, the grain size of ferrite (F) gradually increases while the content of martensite (M) gradually increases, and the grain is refined. During the segmentation process, we also obtained consistent conclusions. The sample was prepared using Mn-Si type dual-phase steel and photographed under laboratory conditions using a metallographic optical microscope.

### 2.2. Validation Materials

To ensure the feasibility of the method, we prepared validation steel and designed an experimental component, Mn-Si DP validation steel, to ensure the feasibility of the experimental segmentation algorithm. Table 1 provides detailed information. We validated the algorithm using the following components to validate the steel. Two-phase steel samples were heat-treated at temperatures of 740 °C, 760 °C, 780 °C, 800 °C, 820 °C, 840 °C, 860 °C, and 900 °C, respectively.

### 2.3. Microstructure Characterization

Metallographic microstructure analysis is a basic and commonly used method. The performance of a material depends on its internal microstructure, which is also the core link of material design. Obtaining the microstructure of materials is a routine task in the study of materials. In metallographic preparation and analysis, metallographic techniques are used to obtain in-depth internal microstructure details of materials at the micrometer and submicrometer scales. Through general metallographic analysis, for microstructure analysis, it is necessary to first obtain an optical photograph of the tissue. The experiment used a normal imaging rate (1000 ns/pixel) to generate high-quality images for constructing training and testing datasets. After grinding and polishing, the sample was rotated at 300 rpm for 20 s. A typical backscattered electron (BSE) image of DP steel contains martensite (M) and austenite (A). It can be observed that most of the grain boundaries were somewhat fuzzy, and the morphological features of austenite in different locations were not consistent, representing a difficulty in the accurate classification. After heat treatment, the sample was processed by electric discharge wire cutting, sandpaper mechanical grinding, and polished with 1.5 μm diamond polishing paste. After polishing, it was cleaned with alcohol and blow-dry and eroded with 4% nitric acid alcohol. Finally, metallographic microscopy (OM) observation was performed.

### 2.4. Dataset Construction

The segmentation method validation set includes two DP images. The recognition method mainly utilizes 40 DP steel images to construct a dataset of depth models, including 15,162 patches for training and 5968 patches for validation.

## 3. Methods

**Figure 1:**
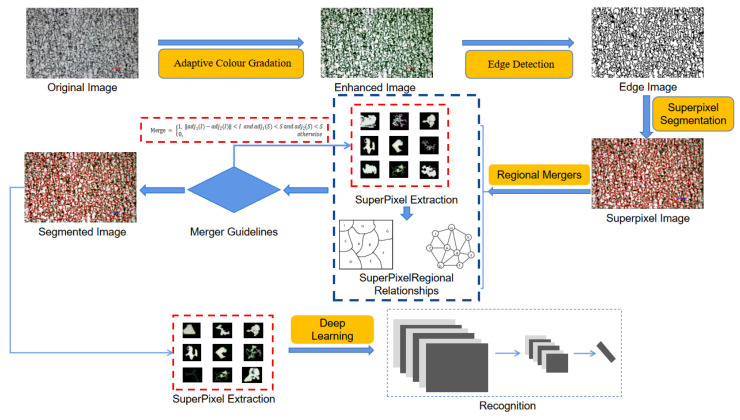
Overall flow chart of the methodology used in this work. The arrows indicate the sequential flow and logical progression of the proposed methodology. The red dashed boxes present the results obtained at different methodological stages, while the blue dashed boxes provide explanations of the key methodological steps.

The section details the metallographic organization segmentation method, DPSS. The optical image segmentation method consists of the following steps: image enhancement, edge detection, superpixel segmentation, and region merging. The region-merging method used here is a new merging algorithm (ERAG) that conforms to the characteristics of optical images. Finally, the metallographic grains are extracted according to the segmented results. Based on the extracted grains, the microstructure is then identified using a deep learning network structure. Figure 1 shows the overall flow chart of the main methods implemented in the work. High-strength duplex steels have a wide range of applications in the automotive manufacturing industry due to their excellent mechanical properties. The study uses the low-alloy dual-phase steel (DP-type base material) dataset as a demonstration material to validate the effectiveness of the proposed method.

### 3.1. Optical Image Segmentation

In an optical microscopy image, the ideal grain interior is a smooth area with distinct grain boundaries between adjacent grains. However, most optical images have complication texture, including noise and false edges. The study proposes a superpixel-based segmentation method to address the above problems. The method is roughly divided into two modules. The first, image pre-processing, focuses on enhancing the overall contrast of the image using the automatic color gradation method. Then, the edge detection method is used to detect the edge information of the grains. In the second step, the superpixel method is used to segment the optical image. Thus, the grains can be extracted. The specific detailed methods are as follows.

#### 3.1.1. Image Pre-Processing

**Figure 2:**
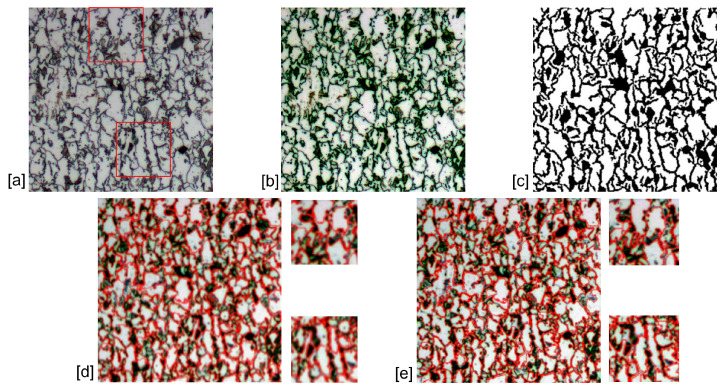
Results of segmentation method step: (**a**) original, (**b**) automatic color gradation, (**c**) edge detection, (**d**) superpixel segmentation (The right panel shows a magnified view of the region outlined in red in the original image), (**e**) region merging (The right panel shows a magnified view of the region outlined in red in the original image).

In general, optical images are affected by some factors, such as material modulation as well as the constraints of the equipment. The grain images under the optical microscope suffer from blurred grain boundaries and similar grayscale between grain interiors and edges. As shown in Figure 2a, the grains and grain boundaries can be initially observed. The microstructure of the specimens consists of grains of ferrite and grain-boundary precipitation of martensite. To increase the accuracy of segmentation, the method of adjusting the color scale is used to increase the contrast between ferrite and martensite, laying the foundation for subsequent segmentation. In general, the color scale of an image is essentially a grayscale distribution of the image. The process of adjusting the color scale is the process of adjusting the grayscale distribution of the image. Here, the image histogram is first used to obtain the overall grayscale distribution. The darkest areas of the image are set to 0, and the brightest areas are set to 255. The pixels in the middle areas are redistributed according to a certain proportion of their gray value. The step increases the overall contrast of the image and achieves an image enhancement effect. The effect is shown in Figure 2b.

Segmenting metallographic grains is a process of grain identification. Edge detection is performed on the metallographic grain images, and edge information is used in the second module for region merging. Here, the boundaries are detected using a flow-based difference-of-Gaussians method [14]. The method calculates the gradient $g(p_{i})$ and normal vector $t(p_{i})$ of each pixel point $p_{i}$ in the image, and then finds the cumulative contrast on both sides of the normal pixel vector. If the contrast between the two sides of the pixel is greater, then the pixel is at the edge. The cumulative contrast is calculated as in Equation (1).

(1) $$H\left(p_{i}\right)=\sum_{s}F\left(p_{s}\right)G_{\sigma_{m}}\left(p_{s}\right),$$

where $p_{s}$ is the s pixels on either side of the pixel normal vector $t(p_{i})$, Specifically, three pixels are taken in the forward direction and three pixels in the backward direction, corresponding to s ∈ [−3, 3]. $G_{\sigma_{m}}$ is a Gaussian filter. $F(p_{s})$ is the contrast function on both sides of the gradient $g(p_{s})$. The step detects most of the grain boundaries and removes most of the noise from inside the grain. However, some boundaries are not closed. The effect is shown in Figure 2c.

#### 3.1.2. Image Segmentation

**Figure 3:**
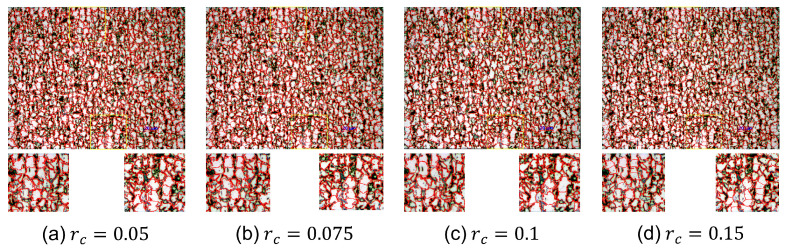
Superpixel segmentation results obtained with different $r_{c}$ values. The yellow boxes in the first row mark the selected local regions, whose magnified views are presented in the second row for clearer comparison.

Superpixels aggregate pixel points with similar characteristics to form prominent representative “pixel” blocks of an image for segmentation. The microstructure of a metallographic grain is made up of multiple grains and precipitations at their boundaries. Common superpixel methods include simple linear iterative clustering (SLIC) algorithm [23], superpixels extracted via energy-driven sampling (SEEDS) algorithm [24], etc. In this study, we adopt the linear spectral clustering (LSC) algorithm proposed by Li and Chen [25] and combine it with an improved region-merging strategy to obtain the final segmentation. In LSC, each pixel is first represented by five normalized variables, (l,α,β,x,y), where l,α,β denote the CIELAB color components and x, y denote the spatial coordinates. The CIELAB space is used because Euclidean distances in this space are approximately perceptually uniform. To construct an explicit feature space for clustering, each of the five variables is further mapped into a cosine-sine pair, yielding a 10-dimensional weighted feature representation. This mapping enables weighted K-means in the transformed space to approximate the normalized-cuts objective. The explicit form of the 10-dimensional feature space is provided in Equation (2),

(2) $$\phi \left(p\right)=\frac{1}{\omega (p)}\left(\begin{array}{cc} C_{c}\cos\frac{\pi }{2}l_{p}, & C_{c}\sin\frac{\pi }{2}l_{p},\\ 2.55C_{c}\cos\frac{\pi }{2}\alpha_{p}, & 2.55C_{c}\sin\frac{\pi }{2}\alpha_{p},\\ 2.55C_{c}\cos\frac{\pi }{2}\beta_{p}, & 2.55C_{c}\sin\frac{\pi }{2}\beta_{p},\\ C_{s}\cos\frac{\pi }{2}x_{p}, & C_{s}\sin\frac{\pi }{2}x_{p},\\ C_{s}\cos\frac{\pi }{2}y_{p}, & C_{s}\sin\frac{\pi }{2}y_{p} \end{array}\right),$$

where *p* is an image pixel point. $C_{c}$ and $C_{s}$ are two parameters controlling color and spatial importance. According to the original LSC formulation, ω(p) is defined as the sum of similarities between pixel *p* and all other pixels, i.e., the node degree in the affinity graph. Thus, ω(p) reflects the overall connectivity of pixel *p*, whereas the relative importance of color and spatial components is determined by $C_{c}$ and $C_{s}$. $r_{c}=C_{s}/C_{c}$ denotes the ratio of $C_{s}$ to $C_{c}$. A smaller $r_{c}$ generally improves boundary adherence, whereas a larger $r_{c}$ tends to produce more regular superpixel shapes. Following the original LSC study, four representative values of $r_{c}=$ (0.05, 0.075, 0.1, and 0.15) were considered to examine the effect of the color-spatial weighting ratio on superpixel segmentation. In our experiments, $r_{c}=$ 0.1 achieved the best segmentation performance for the metallographic images used in this study, as shown in Figure 3. W(p,q) denotes the similarity matrix between the current pixel and the surrounding pixels within its neighborhood. Specifically defined in Equations (3) and (4).

(3) $$\begin{array}{cc} \omega (p) & =\sum_{q\in V}W(p,q)\\ \; & =\omega \left(p\right)\phi \left(p\right)\sum_{q\in V}\omega (q)\phi (q), \end{array}$$

and

(4) $$\begin{array}{cc} W(p,q) & =C_{s}^{2}\left[\cos\frac{\pi }{2}\left(x_{p}-x_{q}\right)+\cos\frac{\pi }{2}\left(y_{p}-y_{q}\right)\right]+C_{c}^{2}\\ \; & [\cos\frac{\pi }{2}\left(l_{p}-l_{q}\right)+2.55(\cos\frac{\pi }{2}\left(\alpha_{p}-\alpha_{q}\right)+\cos\frac{\pi }{2}\\ \; & (\beta_{p}-\beta_{q}))]. \end{array}$$

After the information of the pixel is transformed from color space and coordinate space to feature space, the distance between the pixel point and the cluster center is calculated within the given search area, and the pixel is added to the nearest set of clusters. By iterating continuously and updating the cluster centroids $m_{k}$ during the iterations until the final cluster centroids are not changed, the minimum value $F_{k-m}$ of Equations (5) and (6) is reached.

(5) $$F_{k-m}=\sum_{k=1}^{K}\sum_{p\in \pi_{k}}\omega \left(p\right){∥\phi \left(p\right)-m_{k}∥}^{2},$$

and

(6) $$m_{k}=\frac{\sum_{q\in \pi_{k}}\omega (q)\phi \left(q\right)}{\sum_{q\in \pi_{k}}\omega (q)}.$$

As shown in Figure 2d, after the initial segmentation of the metallographic image by the LSC superpixel algorithm, the superpixel block boundary basically coincides with the grain edge and forms a closed-grain boundary. Unlike most methods, which rely on global segmentation, the superpixel method is based on local segmentation. However, this method suffers from over-segmentation, causing a complete grain boundary to be split into multiple smaller parts. This causes errors in the subsequent measurement of grain size. Therefore, the study proposes a new region-merging model (ERAG) and redefines the merging criteria. The work uses the ERAG method to correct the superpixel block over-segmentation problem.

After the metallic phase image segmentation, it can be initialized into a neighborhood graph model G=(V,E) according to the adjacency of the superpixel blocks, where *V* represents the set of superpixels in the image and *E* represents the relationship between two superpixel blocks. By traversing the *E* within the model, the smallest weight $w_{n}\left(s,t\right)$ is found, and if it satisfies the merging threshold, region *s* and region *t* are merged, and the region neighborhood map is updated. Here, the merge threshold is the optimal empirical value selected according to the edge points contained in most over-segmented regions. The weights between all adjacent regions are recalculated according to the similarity criterion. The above steps are cycled through until no more merging occurs, and the final neighborhood graph model $G^{\prime }=\left(V^{\prime },E^{\prime }\right)$ is returned.

In particular, the textures of the over-segmented grains are similar, and there is no clear grain-boundary information between them. Therefore, the work redefined merging criteria, i.e., the average gray difference I and the edge length S. The average gray level I is calculated in the LAB color space for the color similarity of neighboring objects. To facilitate the calculation, after determining the color space, the three dimensions of the regional color space are converted into one dimension by means of averaging, and the absolute value distance is found for adjacent regions. The regions are judged according to their color similarity. The edge length S is mainly a count of the number of edge points in each region block. Image pre-processing method mentioned in Section 3.1.1. The edge image of the metallographic micrograph is first extracted, and it contains most of the grain-boundary information. If the gray value of a pixel is 0, the pixel is regarded as an edge point, if the gray value is 255, it is regarded as a non-edge point. The edge length of each superpixel block is then calculated from the edge image by counting the corresponding pixels. Finally, adjacent regions are merged according to Equations (7) and (8), where 1 indicates that two regions are merged and 0 indicates that they are not merged.

(7) $$Merge=\left\{\begin{array}{c} 1,m\\ 0,otherwise \end{array}\right.$$

(8) $$\begin{array}{c} m=∥adj_{i}\left(I\right)-adj_{j}\left(I\right)∥<I\;and\\ adj_{i}\left(S\right)<S\;and\;adj_{j}\left(S\right)<S, \end{array}$$

Here, $adj_{i}\left(I\right)$ denotes the mean gray value of the region, with the threshold set to 50, and $adj_{i}\left(S\right)$ denotes the number of edge pixels in the region, with the threshold set to 300. These values were determined empirically for the current dataset. After conditional region merging, the over-segmented grains are effectively combined, and the original grain morphology is largely restored, as shown in Figure 2e.

### 3.2. Identifying Phase Organization

In the work, a deep learning approach is mainly used to identify the microstructure of metal phases. A lightweight convolutional neural network model is constructed to learn the microstructural features of metal phases and identify their phase organization.

**Figure 4:**
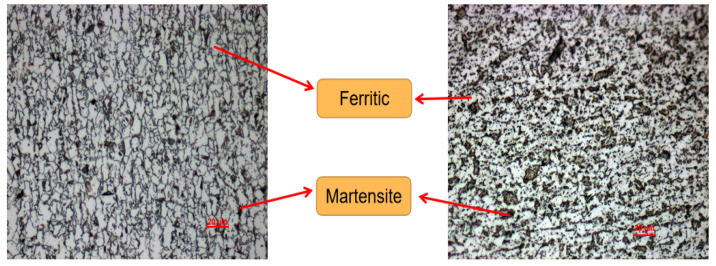
Schematic optical image of the microstructure of steel, ferrite in grain form, martensite precipitated at grain boundaries.

**Figure 5:**
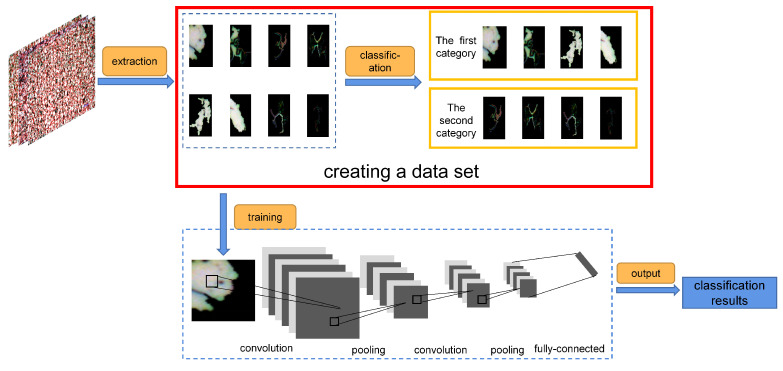
Metallographic microstructure recognition model, a lightweight classification model consisting of two convolutional layers, two pooling layers, and a fully connected layer. The arrows indicate the sequential workflow of the proposed method. The red boxes illustrate the intermediate effects of the classification process, while the yellow boxes present the final recognition results for each class.

Here, a complex texture image recognition and segmentation model based on a superpixel algorithm and deep learning proposed by Han et al. [26] is referenced. As shown in Figure 4, the superpixel-based recognition algorithm mainly distinguishes the two major phases in the metallographic image, namely ferrite and martensite. Each segmentation block can be extracted based on the results of the superpixel segmentation. The feature provides a good basis for creating a training set. Many segmentation blocks can be extracted based on the results of superpixel segmentation and used to create a training set. The extracted segmentation results of the metallographic images are divided into two parts based on their average gray values, and both of them are used as training sets. Because the initial segmentation result is irregular in shape, the method crops out the part of each grain block that can be used for learning and classification. The constructed lightweight convolutional neural network consists of multiple types of layers, including convolutional layers for feature extraction, pooling layers for down-sampling, and a fully connected layer for classification, as shown in Figure 5.

## 4. Results and Discussion

**Figure 6:**
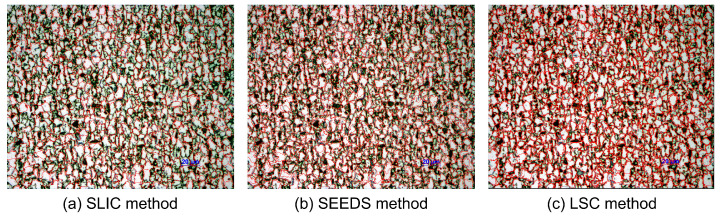
Visual comparison of superpixel segmentation results when K = 2000.

To evaluate the effectiveness of the proposed method for optical image segmentation, experiments were first performed on optical micrographs of low-alloy steel. A comparison among SLIC, SEEDS, and LSC showed that the superpixels produced by LSC adhered more closely to the grain boundaries, resulting in more accurate grain delineation and providing a solid foundation for the subsequent region-merging step, as illustrated in Figure 6. Different segmentation methods were then further compared to demonstrate the advantage of the proposed method.

**Figure 7:**
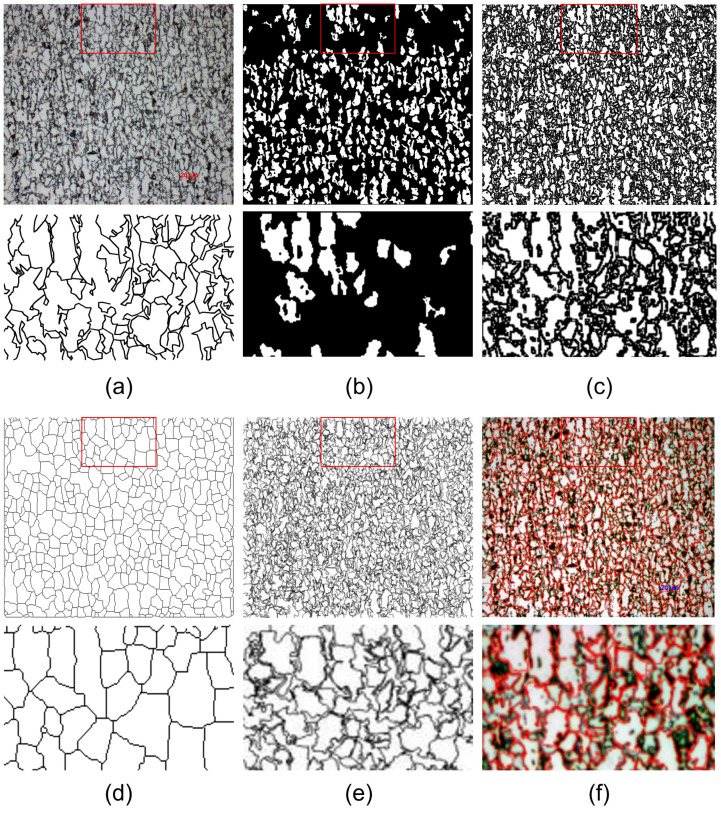
Comparison of grain extraction results for the sample optical image. The red boxes in the first row mark the selected local regions, whose magnified results are presented in the second row for detailed comparison. (**a**) Original image with the expert-annotated reference label for the selected local region, (**b**) result obtained by the modified FDOG method, (**c**) result obtained by the ImageJ method, (**d**) result obtained by the adaptive morphology-based method, (**e**) contour result of the proposed method, (**f**) segmentation result of the proposed method overlaid on the original image. The proposed method preserves grain-boundary information more clearly, produces fewer false edges, and gives the same grain size number of 9 as the manual measurement.

**Figure 8:**
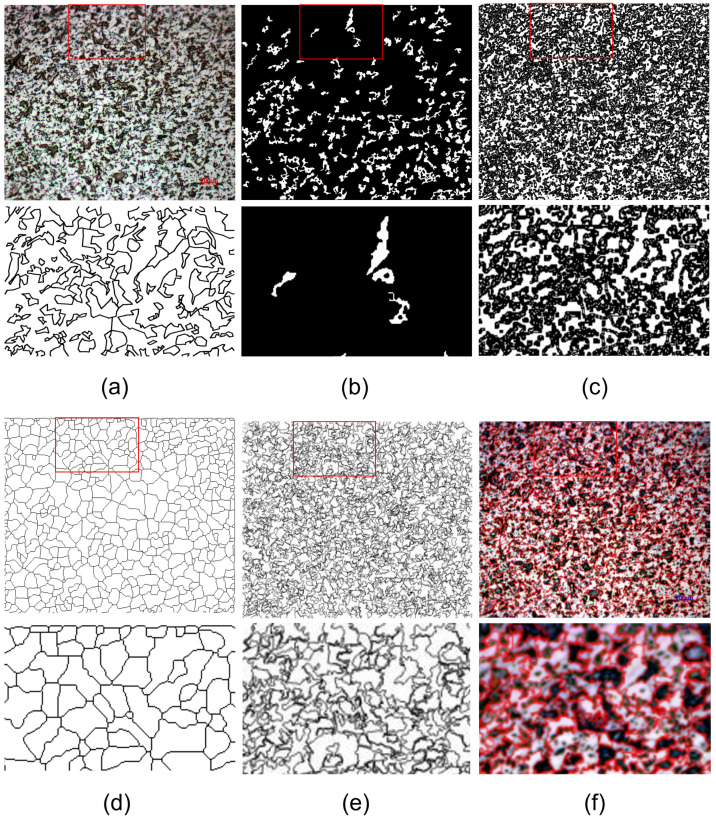
Comparison of grain extraction results for the sample optical image. The red boxes in the first row mark the selected local regions, whose magnified results are presented in the second row for detailed comparison. (**a**) Original image with the expert-annotated reference label for the selected local region, (**b**) result obtained by the modified FDOG method, (**c**) result obtained by the ImageJ method, (**d**) result obtained by the adaptive morphology-based method, (**e**) contour result of the proposed method, (**f**) segmentation result of the proposed method overlaid on the original image. For this metallographic image, martensite is the main segmentation target, and the proposed method extracts the martensitic regions more effectively. The manual measurement gave a grain size number of 10, whereas the proposed method gave a grain size number of 9, indicating that the proposed method achieves results close to expert evaluation.

**Figure 9:**
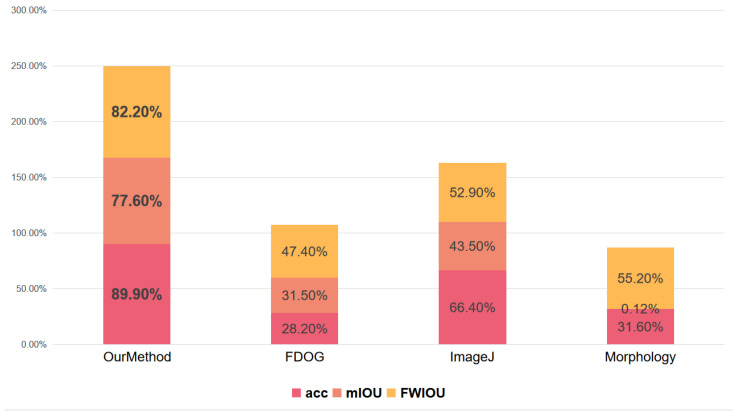
Accuracy (acc), Mean Intersection over Union (mIOU), and Frequency Weighted Intersection over Union (FWIOU) results of the four segmentation methods in Figure 7.

As can be seen from the results in Figure 7 and Figure 8, the segmentation method is effective in segmenting metallographic grains and grain-boundary precipitation. The original image in Figure 7 shows a representative sample of the micrographs used in our experiments and their local enlargements. In the micrograph, most grain boundaries and grains can be observed relatively clearly. In general, the microstructure of the specimen consists of ferrite and bainite. We use the FDOG method, the ImageJ method, and the morphological method to compare the effectiveness of the proposed method. The specific results are shown in Figure 7. Figure 7a presents the original sample image, in which the grain morphology and grayscale distribution can be directly observed. The FDOG result in Figure 7b shows the extracted grain information and its local enlargement obtained using the modified FDOG method. However, this method extracts only limited grain information, and considerable image information is lost. The ImageJ result in Figure 7c shows the grain information extracted using ImageJ software (Version: 1.54f). In this result, the extracted grain-boundary information is excessively cluttered, and the intragranular precipitates are also detected as edges, resulting in incorrect boundary information. This makes subsequent grain measurement more difficult. Figure 7d shows the result of an adaptive image morphology-based method proposed by Siddhartha Banerjee in 2019 for analyzing the sample image. Although this method can extract most of the grain information more completely, it still produces errors in the detection of some small grains. Figure 7e,f present the contour extraction result and the final segmentation result of the proposed method, respectively, as well as the effect of superimposing the segmentation result on the original image. As shown in the figure, there are four advantages of segmentation using the automatic segmentation method: first, the method enhances the overall contrast of the image by reprogramming the grayscale distribution of the optical image. Second, the pixel point features in the image are transformed from low-dimensional to higher-dimensional pixel features with stronger differentiability, which improves the segmentation accuracy of the algorithm. Third, the number of pixels and edge points of each superpixel block is detected to complete the merging of similar superpixel blocks, which finally restores the grain morphology. Fourth, the principle of superpixel segmentation is used to recover most of the weak edge information. In addition to the segmentation comparison graph, we calculated the pixel accuracy (acc), Mean Intersection over Union (mIOU), and Frequency Weighted Intersection over Union (FWIOU) of each segmentation result graph. Based on the calculation results, the average accuracy of the segmentation model proposed in the study is 89%. The specific data are shown in Figure 9.

**Figure 10:**
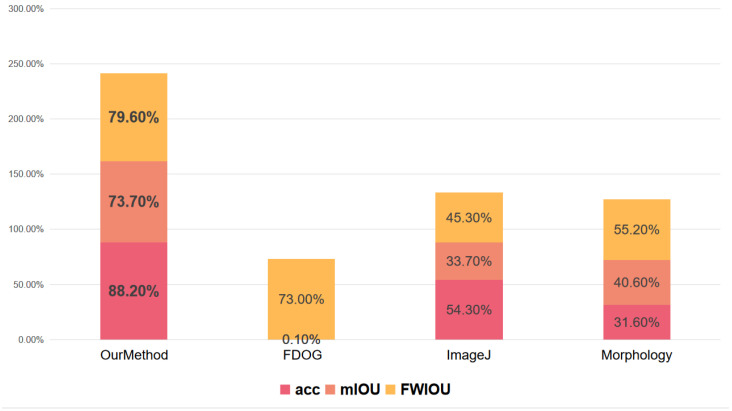
Accuracy (acc), Mean Intersection over Union (mIOU), and Frequency Weighted Intersection over Union (FWIOU) results of the four segmentation Methods in Figure 8.

It can be observed visually that the original image in Figure 8a, unlike the image shown in Figure 7a, the intensity within the grains is not uniform enough, there is more bainite precipitated from the grain boundaries, the grain boundaries are blurred, and the image itself suffers from poor microscopic sampling. The results of the grain boundaries extracted by the four methods are shown in Figure 8. Obviously, the method proposed in the work can identify grain boundaries and extract grains more accurately than the other three methods. The quantitative metrics for each segmentation result are presented in Figure 10.

Based on the above experimental results of the segmentation of optical images, the microstructure of optical images can be identified. The work used microstructure superpixel blocks extracted from steel optical images as the dataset for the recognition classification model. A total of 46,920 microstructure image blocks were extracted from 40 low-alloy steel optical images. In order to illustrate the auto recognition effectiveness, our method is compared with multiple models. The specific experimental results are shown in Table 2. Upon comparison, our model performs better in recognition accuracy. The final classification recognition accuracy can reach 99.91%. Compared with the fully connected models, the convolutional neural network is more suitable for this task because it can better extract local spatial features from the segmented microstructure blocks. The fully connected layers mainly transform the input into vectors and output classification probabilities through weighting and nonlinear operations, whereas convolution extracts localized texture and morphological features through sliding windows. Since the input samples in this study are relatively small segmented image blocks, a lightweight convolutional architecture is sufficient to capture the discriminative information required for classification. Compared with LeNet5 [27], the proposed model has a more compact structure, fewer layers, and only one fully connected layer. This design reduces the number of parameters and improves parameter efficiency. For the relatively small image patches used in this study, a deeper network such as LeNet5 is not necessarily more effective, because deeper feature extraction stages may provide limited additional useful information. In contrast, the lightweight network proposed in this work can better utilize the valid information in the image for classification and recognition, thereby achieving high accuracy with lower computational cost. Since the main purpose of the classification module is to verify the effectiveness of the proposed segmentation method rather than to develop a new deep classification framework, the lightweight model is an appropriate choice in this work.

The percentage of different microstructures in dual-phase steel produces modulated alloys with different characteristics. With different heat treatments and processing of the alloy, the metallographic characteristics will also change, resulting in various phase microstructures. The microstructure composition and morphology of complex metallographic images are diverse, making it difficult to extract useful information. Only by accurately segmenting grains can a reliable basis be provided for material performance analysis.

The superpixel-based segmentation method proposed in this study can extract closed grain contours from dual-phase steel metallographic images with higher accuracy and completeness than the comparison methods. Compared with conventional segmentation methods, the proposed approach shows better adaptability in dealing with blurred grain boundaries, complex microstructural morphologies, and non-uniform grayscale variations within grains. Although certain grayscale variations exist inside the grains and the contrast between grain boundaries and intragranular regions is not always pronounced, the proposed method can still recover the actual grain-boundary positions with relatively high accuracy and obtain more continuous and complete grain contours. This demonstrates that the method has strong segmentation capability for complex metallographic microstructure images and can effectively reduce the under-segmentation and over-segmentation problems commonly encountered in traditional methods. The proposed method can serve as an effective alternative to conventional material image segmentation methods, such as the FDOG method, ImageJ method, and Morphological method. On this basis, when combined with a deep-learning-based recognition method, the proposed framework can further improve the identification accuracy of martensite and ferrite. Because the segmentation results preserve the boundary information and morphological features of the target regions more completely, they provide more reliable inputs for subsequent phase classification, thereby improving the stability and accuracy of automated microstructure recognition. Based on the segmentation and recognition results, further analyses can also be carried out, including grain size statistics, microstructural composition analysis, and the investigation of the relationships among processing parameters, grain size, and microstructural evolution, thus providing a reliable basis for performance evaluation and microstructure control of dual-phase steels.

The experiments in this study were all conducted on metallographic images obtained under a standard 4% Nital etching condition. However, variations in etching severity may affect grain-boundary clarity, phase contrast, and local texture features, which may in turn influence superpixel partitioning and the subsequent region-merging process. In addition, the proposed method still relies on preset parameters, and its robustness and generalization under different sample preparation and imaging conditions have not yet been fully validated. Therefore, future work will extend this framework to other classes of steels with more complex microstructures, such as ferrite-pearlite steels, bainitic steels, martensitic steels, and multiphase steels, in order to evaluate its transferability and practical potential in broader metallographic analysis.

## Figures and Tables

**Table 1:** Verify the experimental composition of steel.

	C	Mn	Si	Fe
DP verification steel 1	0	2	0	98
DP verification steel 2	0	1.5	0	98.5

**Table 2:** Accuracy of metallographic image recognition models. (“FC-1, FC-2, FC-3” denote three fully connected baseline networks with progressively increasing architectural complexity, in which nonlinear activation functions and normalization components are introduced step by step, while LeNet5 is included as a classical convolutional neural network baseline for comparison).

Model	FC-1	FC-2	FC-3	LeNet5	OurModel
Structure	fc1 30 × 27	fc1 30 × 27	fc1 30 × 27	conv2d 5 × 5.6	conv2d 3 × 3.64
	fc2 27 × 20	ReLU	norm	ReLU	norm
	fc3 20 × 2	fc2 27 × 20	ReLU	maxpool2d 2 × 2	ReLU
		ReLU	fc2 27 × 20	conv2d 5 × 5.16	maxpool2d 2 × 2
		fc3 20 × 2	norm	ReLU	conv2d 3 × 3.64
			ReLU	maxpool2d 2 × 2	norm
			fc3 20 × 2	fc1 400 × 120	ReLU
				fc2 120 × 84	maxpool2d 2 × 2
				fc3 84 × 2	fc 256 × 2
Acc	99.74%	99.71%	99.58%	99.73%	99.91%

## Data Availability

The data presented in this study are available on request from the corresponding author due to all data involve a confidential project of Ansteel Group Beijing Research Institute Co., Ltd.
